# Relative contribution of comorbid diseases to health-related quality of life in patients with Parkinson’s disease

**DOI:** 10.1186/s41687-024-00746-4

**Published:** 2024-08-05

**Authors:** Maija-Helena Keränen, Laura Kytövuori, Juha Huhtakangas, Mikko Kärppä, Kari Majamaa

**Affiliations:** 1 Research Unit of Clinical Medicine, University of Oulu, P.O. Box 5000, Oulu, FI-90014 Finland; 2 Medical Research Center Oulu, Oulu University Hospital and University of Oulu, Oulu, Finland; 3 Neurocenter, Oulu University Hospital, P.O. Box 20, Oulu, FI-90029 OYS Finland

**Keywords:** Comorbidity, Generic questionnaire, Older adults, Cohort studies

## Abstract

**Background:**

Multimorbidity is common in elderly people, and one of the major consequences of multimorbidity is low health-related quality of life (HRQoL). The aim of this study was to investigate the frequency of comorbid diseases in patients with Parkinson’s disease (PD) and to analyze their relative importance in HRQoL. The aim was also to examine agreement between the generic 15D questionnaire and the PD-specific Parkinson’s Disease Questionnaire (PDQ-8) to further validate 15D in the evaluation of HRQoL in patients with PD.

**Methods:**

Patients with PD (*N* = 551) filled a questionnaire on comorbid diseases, and the 15D questionnaire yielding a 15-dimensional health profile and a score representing the overall HRQoL. Self-organizing map was used for an unsupervised pattern recognition of the health profiles. Relative importance analysis was used to evaluate the contribution of 16 comorbid diseases to the 15D score. The agreement between 15D and PDQ-8 questionnaires was studied in a subset of 81 patients that were examined clinically.

**Results:**

533 patients (96.7%) reported comorbid diseases. The most affected dimensions in the 15D questionnaire were secretion, usual activities, discomfort and symptoms, and sexual activity. Self-organizing map identified three patterns of health profiles that included patients with high, low or transition HRQoL. The transition subgroup was similar to low HRQoL subgroup in non-motor dimensions. Sixteen comorbid diseases explained 33.7% of the variance in the 15D score. Memory deficit, depression, heart failure, and atrial fibrillation had the highest relative importance. The intraclass correlation coefficient between the generic 15D and the PD-specific PDQ-8 was 0.642 suggesting moderate reliability.

**Conclusions:**

The most marked differences in HRQoL were in the dimensions of secretion, usual activities, and sexual activity. Pattern detection of 15D health dimensions enabled the detection of a subgroup with disproportionately poor HRQoL in non-motor dimensions. The comorbid diseases affecting most to HRQoL were memory deficit and depression. The generic 15D questionnaire can be used in the evaluation of HRQoL in PD patients.

**Supplementary Information:**

The online version contains supplementary material available at 10.1186/s41687-024-00746-4.

## Background

The peak incidence of Parkinson’s disease (PD) is between 70 and 79 years [1] and prevalence is highest between 85 and 89 years being 1.7% for men and 1.2% for women [2]. Clinically PD is characterized by the motor features tremor, bradykinesia, rigidity, and postural instability as well as the non-motor symptoms such as constipation, insomnia and depression. Multimorbidity is common in the older age groups and comorbidity in association of a given disease is an independent predictor of several outcomes such as higher mortality, lower quality of life, higher healthcare cost and psychological distress [3, 4]. The most common chronic diseases in the general elderly population include hypertension, ischemic heart disease, diabetes, osteoarthritis, and chronic lung diseases [5–7]. Comorbid diseases in patients with PD are similar to those in the general population [8–10] and may be more severe [11].

Health-related quality of life (HRQoL) is lower in patients with PD than in the general population [12–14], and previous studies have suggested that HRQoL in PD depends at least on sex [15, 16], age of onset, and disease duration [16]. More severe motor symptoms, moderate or severe depression, and a higher burden of comorbid diseases decrease HRQoL in PD [16–18]. A study using databases of the US Veterans Health Administration showed that coronary heart disease, arthritis, diabetes, chronic low back pain, congestive heart failure and stroke are comorbid diseases that reduce HRQoL in PD [9]. However, the contribution of various comorbid diseases has rarely been evaluated.

Several HRQoL tools have been used in PD including seven generic and ten disease-specific questionnaires [19]. Generic questionnaires cover a broader range of health domains than disease-specific questionnaires and, hence, generic questionnaires capture an individual’s overall well-being better than the disease-specific questionnaires. The generic instruments have the advantage over the disease-specific instruments that they enable comparison between different disorders. Of the generic questionnaires, four are currently recommended for the use in patients with PD [19].

We have collected a population-based cohort of Finnish patients with a clinical diagnosis of PD. The aim of this study was to investigate HRQoL in patients with PD and to compare health profiles between patients with high or low HRQoL, and to analyze the relative importance of comorbid diseases in the quality of life of these patients. We also examined if the generic 15D questionnaire can be used in the evaluation of HRQoL in patients with PD by comparing the 15D scores to those of the disease-specific Parkinson Disease Questionnaire (PDQ-8).

## Subjects and methods

### Patients

Permanent residents in Finland are entitled to full reimbursement for prescription medication in PD or related disorders. To become eligible for reimbursement a clinical diagnosis of PD by a neurologist is required. The Social Insurance Institution of Finland (Kela) maintains a register of patients with reimbursement for PD medication. Kela mailed the study materials to the patients who were residents of the province of North Ostrobothnia, Kainuu, or North Karelia. A blood sample was taken from the participating patients in the local health care center and the sample, and the completed study questionnaires, were mailed to the researchers. A total of 569 patients participated in the study. The proportion of patients living at home was 96.9% (*N* = 534) of whom 354 patients lived independently, and 180 patients had various forms of home care support or services. Seventeen patients were in residential care.

The study questionnaire included a selection of comorbid diseases (Supplementary Table 1), and the generic 15D questionnaire assessing health-related quality of life [20]. In the former questionnaire patients were instructed to report only those diseases that had been diagnosed by a clinician. Exclusions from the analyses (*N* = 18) included 13 patients who left four or more missing items in the 15D questionnaire, one patient who did not respond to the questionnaire on comorbidity, and four patients with missing items in both questionnaires. The agreement between 15D HRQoL and PDQ-8 was analyzed in a subset of 81 patients, who were examined clinically.

### Health-related quality of life questionnaires

The 15D quality of life questionnaire is a generic, multidimensional, standardized, self-administered evaluative tool and has good test–retest reliability, construct validity, and discriminatory power in general populations [20]. The 15D questionnaire comprises five ordinal answer options (the best level, 1; the worst level, 5) that evaluate health status in 15 dimensions including mobility, vision, hearing, breathing, sleeping, eating, speech, elimination, usual activities, mental function, discomfort and symptoms, depression, distress, vitality, and sexual activity [20]. Complete responses to the 15D questionnaire were received from 465 patients and the responses from the remaining 86 patients contained 1–3 missing dimensions (99 dimensions in total, frequency of missingness 1.2%). SPSS syntax for imputing a maximum of 3 dimensions per patient was obtained from the author (www.15d-instrument.net). The syntax also converts the answers to a score (the worst level, 0; the best level, 1) by incorporating a population-based preference weight to each dimension. The converted scores yield a 15-dimensional health profile, and enable the calculation of a single 15D score, which represents the overall HRQoL (full health, 1; dead, 0).

PDQ-8 is an eight-item questionnaire that is specific for Parkinson’s disease [19] and that is one of the four HRQoL tools recommended to be used in studies on PD by the Movement Disorder Society [21]. PDQ-8 contains eight dimensions including mobility, activities of daily of living, emotional well-being, stigma, social support, cognition, communication, and bodily discomfort. A summary index (PDQ-8SI) can be calculated from scores of PDQ-8 and it varies between 0 and 100 (the best level, 0; the worst level, 100) [21].

### Self-organizing map

The 15D questionnaire allows the description of 5^15^ ≈ 3.1 × 10^10^ health profiles. Therefore, self-organizing map (SOM) algorithm was used for an unsupervised pattern recognition. SOM was created from the converted scores of the 15 dimensions in the 15D questionnaire by using the package Numero [22] in R, version 4.2.2 [23]. The SOM radius was set at 3, yielding a map of 40 districts. The algorithm assigns each patient to a single district so that the distances of the patients in the map reflect similarities between the patients. Three subgroups were defined (high HRQoL, *N* = 287; low HRQoL, *N* = 158; transition HRQoL, *N* = 106).

### Statistical methods

Crosstabulation was used to detect significant differences in the frequencies of comorbid diseases between the high-HRQoL and low-HRQoL subgroups. Sixteen diseases with adjusted residuals > 2 in the 2-sided Pearson X^2^ test were then selected for the relative importance analysis [24], where the comorbid diseases were used as independent variables and the 15D score as the response variable. Relative importance in linear regression was assessed by using the averaging over orderings as implemented in the package relaimpo in R [23–25].

Pearson correlation coefficient was used to measure correlation between the 15D and PDQ-8 scores, and paired samples *t* test and Bland-Altman plot were used to analyze agreement. Because the two scoring systems are inverse, we used the proxy variable 1—PDQ-8/100 to obtain scores with the same direction of change (the worst level, 0; the best level, 1). Intraclass correlation coefficient (ICC) was used as an index of reliability. ICC estimates were calculated using SPSS (IBM SPSS Statistics, version 27) based on 2-way mixed-effects model, average measures and absolute agreement.

### Ethics

The study protocol was approved by the Ethics Committee of Oulu University Hospital (EETTMK 51/2017) and by Kela (87/522/2017), and a written informed consent was obtained from the patients or their legal caregivers. Helsinki declaration (2013) was followed in the clinical examinations.

## Results

### Health profiles based on 15D quality of life questionnaire

**Fig. 1:**
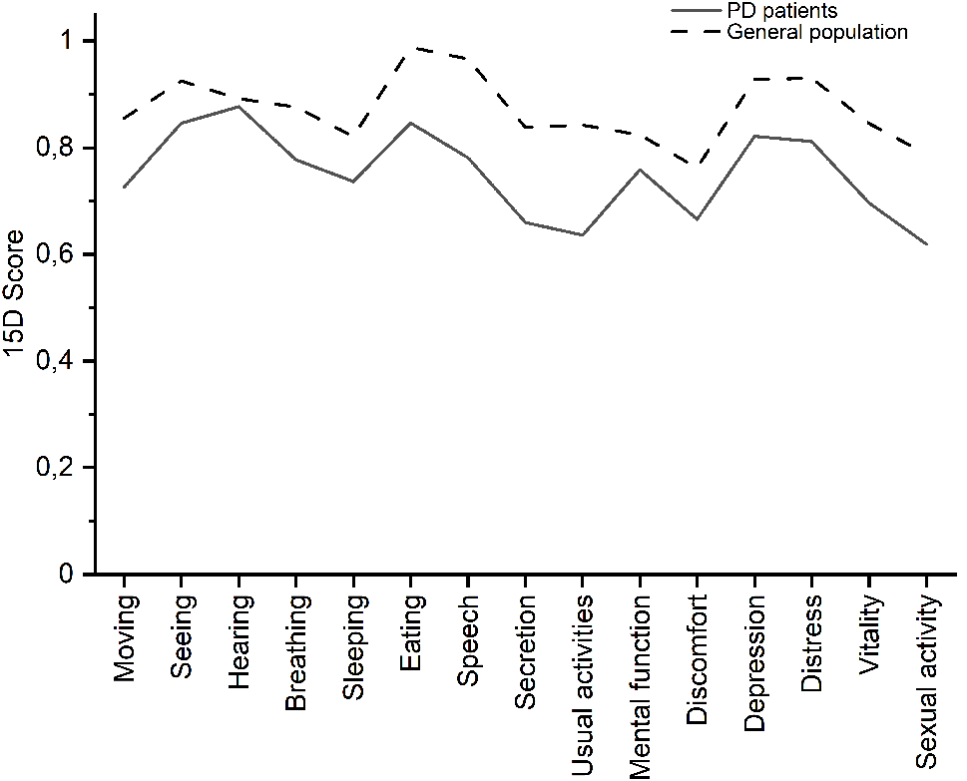
The mean scores of the 15 dimensions in 15D health questionnaire. The profiles are based on responses from patients with PD (*N* = 551) and from 3372 subjects belonging to the Finnish general population [26]

The 551 patients (men, *N* = 334) in the study were 72.0 ± 8.4 years of age (mean ± standard deviation), and the age at onset of PD symptoms was 63.7 ± 9.7 years. The responses of the 551 PD patients to the 15D questionnaire were converted to a score (the worst level, 0; the best level, 1) by incorporating a population-based preference weight to each dimension. The health profile based on these scores showed that the most affected dimensions with scores < 0.70 were secretion, usual activities, discomfort and symptoms, and sexual activity. The scores in the dimensions of secretion, usual activities, and sexual activity were more than 20% lower in the patients with PD than population controls, while less than 10% difference in the mean was found in the dimensions of seeing, hearing, and mental function (Fig. 1).

### Recognition of patterns in health profiles by using self-organizing map algorithm

**Fig. 2:**
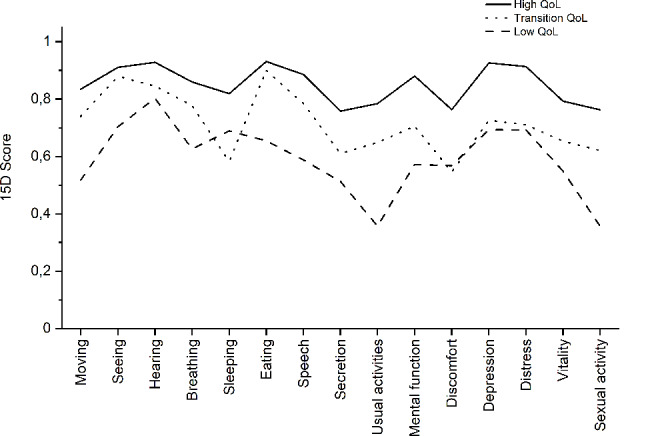
The mean scores of the 15 dimensions in 15D health questionnaire. The profiles are based on responses from patients with PD in high-HRQoL subgroup (*N* = 287), patients in transition HRQoL subgroup (*N* = 106), and patients in low-HRQoL subgroup (*N* = 158)

A self-organizing map was created using the scores of the 15 dimensions in the 551 patients. The SOM displayed three subgroups consisting of 19 districts with high-HRQoL (*N* = 287), 13 districts with low-HRQoL (*N* = 158), and 8 districts with a transition HRQoL (*N* = 106) (Supplementary Figure). The three subgroups differed from each other in age, disease duration and comorbidity count, but not in age at onset (Table 1). The health profiles differed (*p* < 0.001, X^2^ test) between the three subgroups (Fig. 2) except that the scores of the dimensions of hearing, discomfort and symptoms, depression, and distress in the transition HRQoL subgroup were similar to those in the low-HRQoL subgroup (Mann-Whitney U test).

**Table 1:** Clinical features of the PD patients in three SOM subgroups

	HRQoL subgroup			Total
	Transition	High	Low	
Patients, N	106	287	158	551
Age, yr	70.5 ± 7.3	71.2 ± 8.4	74.2 ± 8.4	71.9 ± 8.4
Age at onset, yr	62.9 ± 8.7	63.9 ± 9.7	63.4 ± 10.4	63.6 ± 9.7
Time from diagnosis, yr	7.6 ± 5.1	7.0 ± 4.7	10.4 ± 6.0	8.1 ± 5.4
Disease duration, yr	10.3 ± 6.9	9.1 ± 6.0	13.6 ± 7.0	10.6 ± 6.8
Comorbid diseases, N	4.2 ± 2.3	3.5 ± 2.1	5.0 ± 2.5	4.1 ± 2.3
15D score	0.715 ± 0.061	0.848 ± 0.075	0.585 ± 0.099	0.747 ± 0.14

Values are means ± standard deviations

### Comparison of 15D and PDQ-8 questionnaires

A subset of patients (*N* = 81) also completed the disease-specific PDQ-8 questionnaire. Pearson correlation analysis showed an inverse correlation between the scores (*r* = −0.498, *p* < 0.0001) (PDQ-8 score = 0.78–0.69 × 15D score). Hence, the proxy score 1– PDQ-8/100 was used for further analyses. The proxy and the 15D scores differed by 4% (mean difference − 0.0392 ± 0.1365, *p* < 0.012, paired samples *t* test). The difference was normally distributed (*p* = 0.41, Kolmogorov-Smirnov test) and, therefore, the Bland-Altman plot was analyzed to describe agreement between the two scores. The plot showed that the mean difference of the scores was 0.0064 ± 0.0998 among the 49 patients with the mean of scores ≥ 0.8 (*p* = 0.65 for difference, paired samples *t* test), whereas the mean difference of the scores was − 0.1089 ± 0.1561 (*p* < 0.001) among the 32 patients with the mean of scores < 0.8. Finally, the ICC was calculated to be 0.642 (0.444; 0.770, 95% confidence interval).

### Frequency of comorbid diseases

At least one comorbid disease was reported by 533 patients, while 16 (2.9%) men and two (0.4%) women reported no comorbid diseases. The mean number of comorbid diseases was 4.0 among men and 4.5 among women. Hypertension and osteoarthritis were the most frequent comorbid diseases among women, while prostatic hyperplasia and hypertension were the most frequent comorbid diseases among men (Supplementary Table 2).

### Relative importance of comorbid diseases in determining HRQoL

The scores in the 15 dimensions were then used to calculate a single 15D score that was 0.742 ± 0.146 for men and 0.754 ± 0.129 for women. The 15D score correlated with the age of the patients (Pearson *r* = − 0.23, *p* < 0.001) and duration of disease (*r* = − 0.28, *p* < 0.001), but not with age at onset (*r* = − 0.04, *p* = 0.37).

Sixteen comorbid diseases differed significantly in frequency between the SOM subgroups low-HRQoL and high-HRQoL (adjusted residuals > 2, X^2^ test). The contribution of the 16 comorbid diseases to HRQoL was then examined by using relative importance analysis with the 15D score as the response variable. The 16 comorbid diseases explained 33.7% of the variance in the 15D score of the 551 patients. Memory deficit, depression, heart failure, and atrial fibrillation had the highest relative importance in explaining the variation of the 15D score (Table 2). Among men (*N* = 334) the top four comorbid diseases were memory deficit, depression, retinal disease, and atrial fibrillation and they explained 26.5% of the variance, while the remaining 12 comorbidities explained 11.7%. Among women (*N* = 217) the top four comorbid diseases were memory deficit, heart failure, depression, and bone fractures that explained 23.2% of the variance and the remaining 11 comorbid diseases explained 8.5%. Largest differences between the sexes were found in the importance of retinal disease and peptic ulcer that were more important among men, while bone fractures and sequelae of brain injury were more important among women.

**Table 2:** Relative importance of diseases explaining the variation of the 15D score

	Variance explained (%)		
	All	Men	Women
Memory decline	14.9	16.0	11.2
Depression	4.6	5.3	4.5
Heart failure	2.2	1.3	4.9
Atrial fibrillation	1.8	2.0	1.9
Retinal disease	1.2	3.2	0.4
Stroke	1.1	1.6	0.8
Diabetes mellitus	1.0	1.2	0.8
Peptic ulcer	1.0	1.7	0.5
Prostatic hyperplasia	1.0	0.9	NA
Bone fractures	0.9	0.3	2.7
Sleep apnea	0.8	0.8	0.4
Sequelae of brain injury	0.8	0.4	1.7
Spinal stenosis	0.7	0.8	0.8
Thyroid disease	0.6	1.2	0.5
Myocardial infarction	0.6	0.6	0.4
Rheumatic or autoimmune disease	0.5	0.8	0.3

The lmg algorithm [25] was used to estimate the relative importance of the predictors; *NA* not applicable

## Discussion

We found that HRQoL is lower in patients with PD than in the general elderly Finnish population. The mean 15D score was 0.747, whereas the score is 0.873 in Finns aged 65–74 years and 0.807 in Finns aged 75–84 years [14]. The most marked differences were in the dimensions of secretion, usual activities, and sexual activity. The spectrum of comorbid diseases in PD patients was similar to the spectrum of chronic diseases in the population, and the comorbid diseases affecting most to HRQoL were memory deficits and depression that contributed 19.9% to the variation in HRQoL.

We found that almost all (96.7%) of the patients with PD reported at least one comorbid disease. In the Finnish Health 2011 survey [26] 69% of men and 72% of women older than 75 years reported at least one chronic disease, and a linear interpolation gives the frequency of 60% at age 72 years. Thus, our patients with PD reported a comorbid disease significantly more often than subjects in the population reported a chronic disease. We found that the most common comorbid diseases were hypertension, osteoarthritis, bone fractures, and prostatic hyperplasia. In an elderly Swedish population, the most common disorders have been reported to be hypertension with a prevalence of 38%, dementia with a prevalence of 21%, and heart failure with a prevalence of 18% [27]. Several other studies have also shown that these three conditions are the most common diseases among elderly people in addition to depression, diabetes, prostate disorders, arthritis, and respiratory diseases [6, 7, 18]. These diseases are common also among patients with PD [8, 9], and especially dementia, depression, and bone fractures are more common among PD patients than in the general population [28, 29]. The spectrum of comorbid diseases was similar among our patients with PD, but dementia was rare while memory decline was not uncommon being reported by 19% of the subjects. This discrepancy is probably due to the fact that the most advanced patients did not participate in the study.

The four dimensions that were affected most in the 15D health profile were the function of bladder and bowel (secretion), common activities of daily living (usual activities), physical discomfort and symptoms, and sexual activity. Motor impairment is probably the cause leading to poor scores in common activities of daily living. The profile was similar to that seen in a previous Finnish PD cohort [12] and worse than that in the general population [13]. The 15D score in the population of Health 2011 survey was 0.84 in people older than 75 years, whereas the mean score was 0.75 in our PD patients with a mean age of 72 years. A meta-analysis has shown that PD patients have lower HRQoL in physical function and mental health domains compared to the general population [14]. The 15D questionnaire allows the description of more than 3 × 10^10^ health profiles and, therefore, we employed pattern recognition using SOM algorithm to identify subgroups of health profiles. Rather interestingly, the transition subgroup between high-HRQoL and low-HRQoL subgroups was similar to low-HRQoL subgroup in the dimensions of non-motor symptoms including sleeping, discomfort and symptoms, depression, and distress, but dissimilar in the remaining dimensions.

We found that the mean number of comorbid diseases among PD patients with low HRQoL was 5.4 and that with high HRQoL was 3.5 suggesting that a higher number of comorbid diseases contributes to lower HRQoL. A recent study has shown that a higher burden of comorbidities is associated with decreased HRQoL in PD patients [17], but the contribution of various comorbid diseases was not estimated. Indeed, only a few studies have examined the relative importance of single comorbid diseases to HRQoL among PD patients [29]. We analyzed the relative importance of comorbid diseases on HRQoL and found that depression, memory decline, atrial fibrillation and heart failure had the largest effect on HRQoL. Among men the most considerable comorbid diseases were memory deficit, depression, retinal disease, and atrial fibrillation and among women depression, memory deficit, bone fractures and heart failure. Non-motor more than motor symptoms contribute to both physical and mental HRQoL in early PD [30]. Depression, fatigue, and sensory complaints are the non-motor symptoms affecting most to reduction of HRQoL during the first three years of PD [30–33] followed by non-tremor motor dysfunction and insomnia and anxiety [34]. Non-motor comorbid diseases appear to have the greatest effect on HRQoL in PD.

The 15D questionnaire has been used only occasionally to assess quality of life among patients with PD [12, 35] and, in consequence, the Movement Disorder Society Task Force has rated 15D as a “suggested” but not “recommended” method to be used for patients with PD [19]. We found a good correlation between the 15D and PDQ-8 scores and ICC suggested moderate reliability. A good correlation between 15D and PDQ-8 and a high ICC between 15D and EQ-5D-5 L has also been found among 133 Spanish patients with PD [35]. Differences between the three groups created in the SOM analysis can be considered to present clinically relevant changes. The difference was 0.133 in the mean of 15D score between high-HRQoL and transition HRQoL subgroups and 0.130 between low-HRQoL and transition HRQoL subgroups suggesting that this value can be confidently used as minimal clinically important change (MCIC). Extrapolation of the plot of difference between PDQ-8 and 15D as dependent variable and 15D score as independent variable suggests that the 15D score of 0.13 equals to the mean difference of 0.266 between the scores. This value of MCIC is similar to the minimal detectable change calculated from the Bland-Altman plot suggesting that the two HRQoL questionnaires may be used interchangeably. The conclusion is more valid among subjects with a mean score ≥ 0.80 suggesting that the questionnaires perform equally well at least for patients with high HRQoL.

There are some limitations in our study. The protocol did not favour the participation of patients with the most advanced disease or with cognitive problems, as the questionnaire was mailed to the home address of the patients and as the questionnaire was rather extensive. In consequence, only 3% of the responses were from patients in residential care, while administrative data show that 4% of the Finnish population aged > 65 years were in an institution in 2021 [36]. A detailed analysis of German administrative data has shown that residential care is required for PD patients 3.5-fold more often than for individuals without PD [37]. Comorbid diseases were self-reported, but it was required that only diagnoses made by a medical doctor should be reported. Only selected conditions were studied and some common diseases, such as respiratory diseases, were not included in the questionnaire. The severity of comorbid diseases was not taken into account when assessing the relative importance of comorbidities.

## Conclusions

HRQoL was lower in patients with PD than that in the general elderly population. The most marked differences in HRQoL were in the dimensions of secretion, usual activities, and sexual activity. Pattern detection of the health dimensions enabled the detection of a subgroup with disproportionately low HRQoL in non-motor dimensions. The spectrum of comorbid diseases in PD patients was similar to the spectrum of chronic diseases in the population, and the comorbid diseases affecting most to HRQoL in PD were memory deficits and depression. We found a good correlation between the generic 15D and the PD-specific PDQ-8 scores, and ICC suggested moderate reliability. The generic 15D questionnaire can be used in the evaluation of HRQoL in PD patients.

### Electronic supplementary material

Below is the link to the electronic supplementary material.

Supplementary Material 1

Supplementary Material 2

## Data Availability

All data relevant to the study is stored in a secured online research platform and is available from the corresponding author on reasonable request.

## Abbreviations

PD: Parkinson’s disease
HRQoL: Health-related quality of life
PDQ-8: Parkinson’s Disease Questionnaire-8
PDQ-8SI: PDQ-8 summary index
SOM: Self-organizing map
ICC: Intraclass correlation coefficient
