# Supplementary material for: Relative contribution of comorbid diseases to health-related quality of life in patients with Parkinson’s disease
Source: J Patient Rep Outcomes. 2024 Aug 5;8:84. doi: 10.1186/s41687-024-00746-4 (PMC11300740; doi:10.1186/s41687-024-00746-4)

Supplementary Figure. Self-organizing map created from 15D health profiles of 551 PD patients. Three subgroups were identified. 1, transition HRQoL subgroup; 2, high-HRQoL subgroup; 3, low-HRQoL subgroup


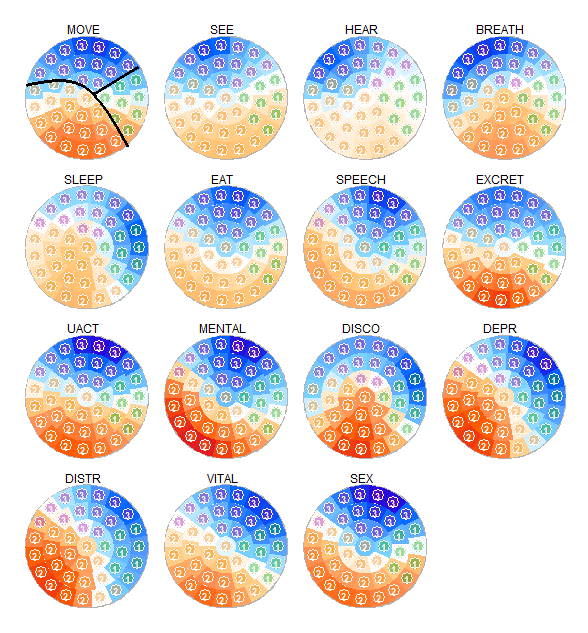

Supplement: Supplementary file 1 — Supplementary Material 1 [file 41687_2024_746_MOESM1_ESM.docx]
