# Supplementary material for: Relative contribution of comorbid diseases to health-related quality of life in patients with Parkinson’s disease
Source: J Patient Rep Outcomes. 2024 Aug 5;8:84. doi: 10.1186/s41687-024-00746-4 (PMC11300740; doi:10.1186/s41687-024-00746-4)
Supplement: Supplementary file 2 — Supplementary Material 2 [file 41687_2024_746_MOESM2_ESM.docx]

Supplementary Table 1. Comorbid diseases included in the questionnaire mailed to the patients.

| Vascular diseases | Hypertension |
| --- | --- |
|  | Atrial fibrillation |
|  | Coronary artery disease |
|  | Myocardial infarction |
|  | Heart failure |
|  | Peripheral vascular disease |
| Neurological diseases/conditions | Memory decline |
|  | Stroke |
|  | Migraine |
|  | Epilepsy |
|  | Sequelae of brain injury |
|  | Sleep apnea |
| Mental disorders | Depression |
|  | Other mental disorder |
| Musculoskeletal diaseases | Osteoarthritis |
|  | Other articular disease |
|  | Sciatica |
|  | Spinal stenosis |
|  | Osteoporosis |
|  | Gout |
|  | Other rheumatic or autoimmune disease |
|  | Bone fractures |
| Diseases of the skin | Atopic dermatitis |
|  | Psoriasis |
| Diseases of the eye | Macular degeneration |
|  | Diabetic macular disease |
|  | Glaucoma |
| Endocrine diseases | Diabetes mellitus type 1 |
|  | Diabetes mellitus type 2 |
|  | Hypothyroidism |
|  | Hyperthyroidism |
| Neoplasms | Skin cancer |
|  | Cancer (other than skin cancer) |
| Diseases of the digestive system | Coeliac disease |
|  | Peptic ulcer |
| Diseases of the genitourinary system | Prostatic hyperplasia |

Supplementary Table 2. Frequencies of comorbid diseases among 551 patients with PD.

|  | All  N (%) | Men  N (%) | Women  N (%) |
| --- | --- | --- | --- |
| Hypertension | 250 (45.4) | 145 (43.4) | 105 (48.4) |
| Osteoarthritis | 193 (35.0) | 92 (27.5) | 101 (46.5) |
| Bone fractures | 167 (30.3) | 84 (25.1) | 83 (38.2) |
| Prostatic hyperplasia | 152 (27.6) | 152 (45.5) | NA |
| Sciatica | 120 (21.8) | 65 (19.5) | 55 (25.3) |
| Memory decline | 104 (18.9) | 74 (22.2) | 30 (13.8) |
| Coronary artery disease | 95 (17.2) | 65 (19.5) | 30 (13.8) |
| Depression | 95 (17.2) | 49 (14.7) | 46 (21.2) |
| Thyroid disease^a^ | 85 (15.4) | 34 (10.2) | 51 (23.5) |
| Atrial fibrillation | 78 (14.2) | 49 (14.7) | 29 (13.4) |
| Diabetes mellitus^b^ | 77 (14.0) | 50 (15.0) | 27 (12.4) |
| Spinal stenosis | 75 (13.6) | 44 (13.2) | 31 (14.3) |
| Stroke | 66 (12.0) | 34 (10.2) | 32 (14.7) |
| Retinal disease^c^ | 65 (11.8) | 29 (8.7) | 36 (16.6) |
| Migraine | 62 (11.3) | 22 (6.6) | 40 (18.4) |
| Cancer^d^ | 97 (17.6) | 60 (18.0) | 37 (17.0) |
| Osteoporosis | 54 (9.8) | 7 (2.1) | 47 (21.7) |
| Sleep apnea | 53 (9.6) | 40 (12.0) | 13 (6.0) |
| Heart failure | 52 (9.4) | 25 (7.5) | 27(12.4) |
| Atopic dermatitis | 38 (6.9) | 23 (6.9) | 15 (6.9) |
| Rheumatic disease^e^ | 37 (6.7) | 15 (4.5) | 22 (10.1) |
| Gout | 36 (6.5) | 23 (6.9) | 13 (6.0) |
| Peripheral vascular disease | 35 (6.4) | 18 (5.4) | 17 (7.8) |
| Peptic ulcer | 33 (6.0) | 18 (5.4) | 15 (6.9) |
| Glaucoma | 32 (5.8) | 17 (5.1) | 15 (6.9) |
| Myocardial infarction | 30 (5.4) | 21 (6.3) | 9 (4.1) |
| Psoriasis | 18 (3.3) | 14 (4.2) | 4 (1.8) |
| Brain injury | 15 (2.7) | 7 (2.1) | 8 (3.7) |
| Coeliac disease | 14 (2.5) | 9 (2.7) | 5 (2.3) |
| Epilepsy | 12 (2.2) | 10 (3.0) | 2 (0.9) |

^a^includes hypothyroidism and hyperthyroidism

^b^includes diabetes mellitus type 1 and 2

^c^includes diabetic retinopathy and age-related macular degeneration

^d^includes any current or previous malignant disease

^e^includes other autoimmune diseases
